# Supplementary material for: Analysis of PPARγ Signaling Activity in Psoriasis
Source: Int J Mol Sci. 2021 Aug 10;22(16):8603. doi: 10.3390/ijms22168603 (PMC8395241; doi:10.3390/ijms22168603)
Supplement: Supplementary file 1 [file ijms-22-08603-s001.zip › Supplemental materials_Analysis of PPARg signaling activity in psoriasis/Pathway models/Models images and html files/Differentiation of psoriatic T cells/6333.html]

IL6R


# Protein IL6R

|  |  |
| --- | --- |
| URN | urn:agi-llid:3570 |
| Total Entities | 0 |
| Connectivity | 2773 |
| Name | IL6R |
| Description | interleukin 6 receptor |
| Notes | This gene encodes a subunit of the interleukin 6 (IL6) receptor complex. Interleukin 6 is a potent pleiotropic cytokine that regulates cell growth and differentiation and plays an important role in the immune response. The IL6 receptor is a protein complex consisting of this protein and interleukin 6 signal transducer (IL6ST/GP130/IL6-beta), a receptor subunit also shared by many other cytokines. Dysregulated production of IL6 and this receptor are implicated in the pathogenesis of many diseases, such as multiple myeloma, autoimmune diseases and prostate cancer. Alternatively spliced transcript variants encoding distinct isoforms have been reported. A pseudogene of this gene is found on chromosome 9.[provided by RefSeq, May 2011] |
| Primary Cell Localization | Plasma membrane |
| Class | Receptor |

---

|  |  |
| --- | --- |
| Pathway | AHR Signaling in Th17 Cells Function |
|  | Th17-Cell Differentiation |
|  | Negative Acute Phase Proteins Synthesis |
|  | Positive Acute Phase Proteins Synthesis |
|  | Vascular Endothelial Cell Activation by Cytokines |
|  | Adipokines Production by Adipocyte |
|  | IL6 Expression Targets -> Nociception |
|  | Megakaryocyte -> Platelet Precursor Cell Surface Expression Markers |
|  | Cardiomyocyte Hypertrophy |
|  | Hypertrophic Cardiomyopathy |
|  | Proteins Involved in Pulmonary Hypertension |
|  | Lymphocyte Mediated Myocardial Injury in Myocarditis |
|  | SOCS in Negative Feedback Regulation in Myocarditis |
|  | T-Cells Differentiation Block in Psoriasis |
|  | Th17-Cell Function in Systemic Lupus Erythematosus |
|  | Vitiligo |
|  | Humoral Immunity in Vitiligo |
|  | Adipokines and Cytokines in Insulin Resistance in Skeletal Muscles |
|  | Proteins Involved in Insulin Resistance |
|  | Adiponectin Synthesis Declines in Insulin Resistance |
|  | Insulin Resistance in Hepatocytes |
|  | Proteins Involved in non-Alcoholic Fatty Liver Disease |
|  | Oxidation of Fatty Acid in non-Alcoholic Fatty Liver Disease |
|  | Thyroid Hormones in Adipose Tissue Metabolism |
|  | Graves Ophthalmopathy |
|  | Adipokines Production by Adipocyte Impaired in Obesity |
|  | Metastatic Colorectal Cancer |
|  | Th17-Cell Activation in Crohn's Disease |
|  | Diffuse Large-B-Cell Lymphoma |
|  | Diffuse Large-B-Cell Lymphoma ABC Subtype |
|  | Mantle Cell Lymphoma |
|  | IL6/IGF1/VEGFA Signaling in Multiple Myeloma |
|  | Multiple Myeloma |
|  | Proteins Involved in Multiple Myeloma |
|  | Airway Smooth Muscle Cell High Rate Proliferation |
|  | Th17-Cell Differentiation in Asthma |
|  | Mucin Production in Goblet Airway Epithelial Cells |
|  | Osteoclast Activation in Gout |
|  | Neutrophil Recruitment in Sinovium in Gout |
|  | Th17-Cell and Th1 Immune Responsein Psoriatic Arthritis |
|  | Osteoclast Activation in Psoriatic Arthritis |
|  | Synovial Fibroblast Proliferation in Rheumatoid Arthritis |
|  | Osteoclast Activation in Rheumatoid Arthritis |
|  | Proteins Involved in Rheumatoid Arthritis |
|  | Proteins Involved in Systemic Scleroderma |
|  | Skin Fibrosis |
|  | Th17-Cell Numbers Reduction in HIV |
|  | Diabetes Induced Periodontitis |
|  | Drugs Targets in Autistic Disoreder (Hypothesis) |
|  | IL6 and Insulin Resistance in Muscular Dystrophies |
|  | IL6 Promotes Inflammation in Muscular Dystrophies |
|  | Bone Loss in Osteoporosis |
|  | Genes with Mutations Associated with Rheumatoid Arthritis |
|  | Proteins Involved in Glioma |
|  | Glioma Stem Cell Program Activation |
|  | IL6/IL12 Signaling Activates Immune System in Multiple Sclerosis |
|  | Proteins Involved in Endometriosis |
|  | IL6/JAK/STAT3 Signaling in Cholesteatoma |
|  | Cholesteatoma |
|  | Prostate Cancer |
|  | Proteins Involved in Prostate Cancer |
|  | Proteins Involved in Age-Related Macular Degeneration |
|  | Cytokines Trigger Otitis Media |
|  | Endothelial Cell Dysfunction in Glomerulonephritis |
|  | Endothelial Cell Dysfunction in Pyelonephritis |
|  | Receptors and Adaptor Proteins Activated in Cancer |
|  | Proteins with Altered Expression in Cancer-Associated Sustaining of Proliferative Signaling |
|  | STAT3 and NFkB Activate Inflammation-Induced Tumorigenesis |
|  | IL23A and IL17A Provoke Cancer-Associated Inflammation |
|  | STAT3 Inhibits Maturation of Dendritic Cells and Facilitates Cancer Progression |
|  | Epithelial to Mesenchymal Transition in Cancer: Overview |
|  | Cancer Cells Inhibit Adipocyte Differentiation |
|  | Skeletal Muscle Wasting in Cancer Cachexia |
|  | Myeloid Derived Suppressor Cells in Cancer Immune Escape |
|  | IDO1 in Cancer Immune Escape |
|  | IL6R -> CEBP/ELK/SRF Signaling |
|  | IL6R -> STAT Signaling |
|  | IL6 Expression Targets |
|  | FOXO1 Signaling |
|  | PPAR Psoriasis |
|  | Model of PPARG signaling in psoriasis |
|  | 1\_Differentiation of psoriatic T cells |
|  | 2\_The role of endothelial cells in pyelonephritis |
|  | 2\_Cardiomyocyte hypertrophy |
|  | 1\_Th2 cell response in asthma |
|  | 3\_1\_Mucus accumulation in asthma |
|  | 4\_Mucin hyperproduction in goblet and mucous cells in COPD |
|  | 1\_Defects in response to pathogens in the gut promotes inflammation in Crohn's disease |
|  | 1\_Inflammatory mediators increase the production of hepcidin (HAMP) |
|  | 4\_Inflammatory mediators inhibit GATA signaling in erythropoiesis |
|  | 4\_2\_Insulin resistance: inflammation-related insulin resistance |
|  | 4\_3\_Insulin resistance: FFA-related insulin resistance |
|  | Differentiation of psoriatic T cells |

---

|  |  |
| --- | --- |
| Group | Genes with Mutations Associated with Obesity |
|  | Genes with Mutations in GWA Studies of Asthma |

---

|  |  |
| --- | --- |
| MedScan ID | 3570 |

---

|  |  |
| --- | --- |
| LocusLink ID | 3570 |
|  | 16194 |
|  | 24499 |

---

|  |  |
| --- | --- |
| Alias | interleukin IL6 receptor subunit gp80 |
|  | interleukin IL6R alpha |
|  | IL-6 receptor subunit alpha |
|  | IL6R |
|  | interleukin IL-6R I |
|  | interleukin IL6 receptor, alpha subunit |
|  | IL-6R 1 |
|  | interleukin IL6 receptors |
|  | interleukin 6 receptor, alpha |
|  | IL6A\_HUMAN |
|  | IL-6R subunit alpha |
|  | interleukin-6 receptor subunit alpha |
|  | gp80 subunit IL6 receptor |
|  | MGC104991 |
|  | IL6RA |
|  | PIR:I53394 |
|  | sIL-6R gp80 |
|  | IL6RQ |
|  | IL6R alpha |
|  | Soluble interleukin-6 receptors |
|  | interleukin IL6A\_HUMAN |
|  | sIL-6Rs |
|  | interleukin IL6 receptor |
|  | interleukin IL6RA |
|  | MGC30256 |
|  | membrane glycoprotein 80 |
|  | IL6A |
|  | IL6 receptors |
|  | interleukin IL6R |
|  | interleukin 6 receptor alpha subunit |
|  | interleukin IL-6R 1 |
|  | Soluble interleukin-6 receptor |
|  | IL6 receptor |
|  | IL6Q |
|  | IL6 receptor subunit gp80 |
|  | interleukin 6 receptors |
|  | interleukin IL6 receptor gp80 subunit |
|  | gp80 subunit IL6 receptors |
|  | sIL-6R |
|  | IL6 receptor gp80 subunit |
|  | CD126 |
|  | interleukin IL6A |
|  | CD126 antigen |
|  | IL-6R I |
|  | interleukin IL6 Receptor alpha |
|  | OTTHUMP00000034320 |
|  | IL6 Receptor alpha |
|  | interleukin 6 receptor |
|  | gp80 |
|  | IL-6RA |
|  | IL-6R-1 |
|  | IL-6R |
|  | IL-6R-alpha |
|  | IL-6 receptor alpha chain |
|  | IL6R1 |
|  | OTTHUMP00000217870 |
|  | interleukin 6 receptor, alpha subunit |
|  | Interleukin-6 receptor alpha |

---

|  |  |
| --- | --- |
| GO ID | 0070119 |
|  | 0004897 |
|  | 0019955 |
|  | 0004896 |
|  | 0019899 |
|  | 0008083 |
|  | 0042164 |
|  | 0005143 |
|  | 0045519 |
|  | 0019981 |
|  | 0004915 |
|  | 0005138 |
|  | 0042803 |
|  | 0006953 |
|  | 0070120 |
|  | 0019221 |
|  | 0050829 |
|  | 0031018 |
|  | 0097191 |
|  | 0002384 |
|  | 0070102 |
|  | 0002548 |
|  | 0051135 |
|  | 0002827 |
|  | 0042104 |
|  | 0010536 |
|  | 0008284 |
|  | 0032722 |
|  | 0072126 |
|  | 0032729 |
|  | 0032755 |
|  | 0002690 |
|  | 0050671 |
|  | 0032816 |
|  | 0045669 |
|  | 0050731 |
|  | 0048661 |
|  | 0042531 |
|  | 0034097 |
|  | 0016324 |
|  | 0016323 |
|  | 0070110 |
|  | 0009897 |
|  | 0005576 |
|  | 0043514 |
|  | 0070743 |
|  | 0005896 |
|  | 0005886 |
|  | 0043235 |
|  | 0048589 |
|  | 0032966 |
|  | 0043410 |
|  | 0009986 |
|  | 0005615 |
|  | 0016021 |
|  | 0016020 |
|  | 0032809 |
|  | 0051092 |
|  | 0010469 |
|  | 0051591 |
|  | 0045471 |
|  | 0010332 |
|  | 0051384 |
|  | 0032496 |
|  | 0043434 |
|  | 0050830 |
|  | 0032717 |
|  | 0002446 |
|  | 0042517 |
|  | 0016049 |
|  | 0046427 |
|  | 0042981 |
|  | 0045768 |
|  | 0004872 |
|  | 0007165 |
|  | 0005102 |
|  | 0008283 |
|  | 0007166 |
|  | 0006955 |
|  | 0007275 |
|  | 0004907 |
|  | 0005887 |

---

|  |  |
| --- | --- |
| KEGG ID | hsa:3570 |
|  | mmu:16194 |
|  | rno:24499 |

---

|  |  |
| --- | --- |
| MedLine Reference | 10331011 |
|  | 10631556 |
|  | 11306811 |
|  | 12604335 |
|  | 2174054 |
|  | 9870869 |
|  | 10066782 |
|  | 10462375 |
|  | 10785334 |
|  | 10982829 |
|  | 11884403 |
|  | 12002769 |
|  | 12008038 |
|  | 12079407 |
|  | 12372336 |
|  | 12412787 |
|  | 12414855 |
|  | 12419823 |
|  | 12445803 |
|  | 12461182 |
|  | 12472176 |
|  | 12477932 |
|  | 12553038 |
|  | 12553555 |
|  | 12574959 |
|  | 12633863 |
|  | 12643274 |
|  | 12748171 |
|  | 12748876 |
|  | 12789531 |
|  | 1281789 |
|  | 12829785 |
|  | 12832423 |
|  | 12857602 |
|  | 1381187 |
|  | 14557255 |
|  | 14592826 |
|  | 14718574 |
|  | 15041717 |
|  | 15063602 |
|  | 15100312 |
|  | 15166129 |
|  | 15213442 |
|  | 15297310 |
|  | 15456942 |
|  | 15561970 |
|  | 15562008 |
|  | 15579373 |
|  | 15641051 |
|  | 15816827 |
|  | 15837717 |
|  | 15895091 |
|  | 16034137 |
|  | 16127269 |
|  | 16372246 |
|  | 16557588 |
|  | 16794539 |
|  | 16912152 |
|  | 16973585 |
|  | 1872801 |
|  | 1889804 |
|  | 2112585 |
|  | 2157217 |
|  | 2261637 |
|  | 2788034 |
|  | 3136546 |
|  | 8056053 |
|  | 8265649 |
|  | 8428753 |
|  | 8467812 |
|  | 9169421 |
|  | 9373149 |
|  | 9927325 |
|  | 11017875 |
|  | 15284182 |
|  | 15489334 |
|  | 16270750 |
|  | 16710414 |
|  | 17310346 |
|  | 17357077 |
|  | 2529343 |
|  | 8125298 |
|  | 10349636 |
|  | 10521784 |
|  | 11042159 |
|  | 11073809 |
|  | 11076861 |
|  | 11197687 |
|  | 11217851 |
|  | 11275690 |
|  | 11937300 |
|  | 12370259 |
|  | 12413742 |
|  | 12429018 |
|  | 12466851 |
|  | 12643284 |
|  | 1312474 |
|  | 14960573 |
|  | 15073037 |
|  | 15191552 |
|  | 15457342 |
|  | 16141072 |
|  | 16141073 |
|  | 16435023 |
|  | 17202339 |
|  | 2051018 |
|  | 7621883 |
|  | 8392875 |
|  | 8477802 |
|  | 8499630 |
|  | 8676083 |
|  | 8770556 |
|  | 8797792 |
|  | 8836549 |
|  | 9034153 |
|  | 9324354 |
|  | 9659221 |
|  | 9708822 |
|  | 9743535 |
|  | 9874564 |
|  | 11171594 |
|  | 11448096 |
|  | 14751512 |
|  | 15084893 |
|  | 7814800 |

---

|  |  |
| --- | --- |
| Organism | Homo sapiens {Organism urn:agi-taxid:9606} |
|  | Mus musculus {Organism urn:agi-taxid:10090} |
|  | Rattus norvegicus {Organism urn:agi-taxid:10116} |
|  | Mus musculus |
|  | Homo sapiens |
|  | Rattus norvegicus |

---

|  |  |
| --- | --- |
| Mouse chromosome position | 3 39.19 cM |
|  | 3 42.1 cM |

---

|  |  |
| --- | --- |
| OMIM ID | 147880 |
|  | 614752 |
|  | 614689 |

---

|  |  |
| --- | --- |
| Rat chromosome position | 2q34 |

---

|  |  |
| --- | --- |
| Hugo ID | 6019 |
|  | HGNC:6019 |

---

|  |  |
| --- | --- |
| Human chromosome position | 1q21.3 |
|  | 1q21 |

---

|  |  |
| --- | --- |
| Swiss-Prot Accession | A0N0L5 |
|  | P08887 |
|  | A0A087WTB5 |
|  | P08887.1 |
|  | A0A0G2JGF1 |
|  | Q3URV7 |
|  | P22272 |
|  | P22272.2 |
|  | P22273.2 |
|  | A8KAE8 |
|  | B2R6V4 |
|  | Q16202 |
|  | Q53EQ7 |
|  | Q5FWG2 |
|  | Q5VZ23 |
|  | G3V8T6 |
|  | P22273 |
|  | IL6RA\_HUMAN |
|  | IL6RA\_MOUSE |
|  | A0N0L5\_HUMAN |
|  | A8KAE8\_HUMAN |
|  | Q3URV7\_MOUSE |

---

|  |  |
| --- | --- |
| PIR ID | A41242 |
|  | JL0144 |
|  | JL0145 |

---

|  |  |
| --- | --- |
| GenBank ID | NC\_000001 |
|  | NM\_001206866 |
|  | NP\_001193795 |
|  | XM\_017001199 |
|  | XP\_016856688 |
|  | XM\_006711298 |
|  | XP\_006711361 |
|  | XM\_017001200 |
|  | XP\_016856689 |
|  | NM\_000565 |
|  | NP\_000556 |
|  | NM\_181359 |
|  | NP\_852004 |
|  | XM\_005245139 |
|  | XP\_005245196 |
|  | XM\_017001201 |
|  | XP\_016856690 |
|  | XM\_005245140 |
|  | XP\_005245197 |
|  | XM\_006711299 |
|  | XP\_006711362 |
|  | NG\_012087 |
|  | AF104230 |
|  | AL162591 |
|  | CH471121 |
|  | EAW53199 |
|  | EAW53200 |
|  | EAW53201 |
|  | CS068586 |
|  | CAI84624 |
|  | EF064723 |
|  | ABK41906 |
|  | AA035377 |
|  | AK223582 |
|  | BAD97302 |
|  | AK293013 |
|  | BAF85702 |
|  | AK312730 |
|  | BAG35601 |
|  | BC035327 |
|  | BC089410 |
|  | AAH89410 |
|  | BC132684 |
|  | AAI32685 |
|  | BC132686 |
|  | AAI32687 |
|  | BM911928 |
|  | S72848 |
|  | AAC60635 |
|  | X12830 |
|  | CAA31312 |
|  | X58298 |
|  | CAA41231 |
|  | P08887 |
|  | NC\_000069 |
|  | NM\_001310676 |
|  | NP\_001297605 |
|  | NM\_010559 |
|  | NP\_034689 |
|  | AC102392 |
|  | CH466547 |
|  | EDL15180 |
|  | AK020663 |
|  | BAB32168 |
|  | AK046337 |
|  | AK082270 |
|  | AK141197 |
|  | BAE24580 |
|  | BC038306 |
|  | BQ033304 |
|  | X51975 |
|  | CAA36237 |
|  | X51976 |
|  | CAA36238 |
|  | X53802 |
|  | CAA37810 |
|  | P22272 |
|  | NC\_005101 |
|  | XM\_006232588 |
|  | XP\_006232650 |
|  | XM\_006232587 |
|  | XP\_006232649 |
|  | XM\_008761127 |
|  | XP\_008759349 |
|  | NM\_017020 |
|  | NP\_058716 |
|  | XM\_017590628 |
|  | XP\_017446117 |
|  | AC\_000070 |
|  | AABR07012144 |
|  | AABR07072754 |
|  | AAHX01018614 |
|  | AAHX01018615 |
|  | AAHX01018616 |
|  | AAHX01018617 |
|  | AC094121 |
|  | AC128343 |
|  | CH473976 |
|  | EDM00609 |
|  | CB778659 |
|  | M58587 |
|  | AAA41431 |
|  | P22273 |
|  | NC\_018912 |
|  | AMYH02001924 |
|  | AMYH02001925 |
|  | HQ258435 |
|  | ADR83189 |
|  | HQ258616 |
|  | ADR83368 |
|  | XM\_006501045 |
|  | XP\_006501108 |
|  | AC\_000025 |
|  | AAHY01028983 |
|  | AAHY01028984 |
|  | AAHY01028985 |
|  | AAHY01028986 |
|  | AAHY01028987 |
|  | AAHY01028988 |
|  | AAHY01028989 |
|  | BC019388 |
|  | BC148583 |
|  | AAI48584 |
|  | AC\_000133 |
|  | ABBA01049484 |
|  | ABBA01049485 |
|  | ABBA01049486 |
|  | ABBA01049487 |
|  | XM\_005245138 |
|  | XP\_005245195 |
|  | XM\_006232586 |
|  | XP\_006232648 |
|  | XM\_006232585 |
|  | XP\_006232647 |
|  | XM\_006232589 |
|  | XP\_006232651 |
|  | AABR06018833 |
|  | AABR06018834 |
|  | CAH72853 |
|  | NT\_004487 |
|  | NW\_001838529 |
|  | NW\_925683 |
|  | AC\_000044 |
|  | NT\_039240 |
|  | NW\_001030721 |
|  | Q3URV7 |
|  | NW\_047626 |
|  | NW\_001084805 |
|  | Q5FWG2 |
|  | NW\_000191 |
|  | Q5VZ23 |
|  | Q53EQ7 |
|  | 204922 |
|  | 729832 |
|  | 119573584 |
|  | 119573585 |
|  | 124343 |
|  | 124375838 |
|  | 124375840 |
|  | 32581 |
|  | 33846 |
|  | 55663065 |
|  | 58476958 |
|  | 62820287 |
|  | 62898896 |
|  | 633863 |
|  | 119573586 |
|  | 123788393 |
|  | 124344 |
|  | 12861314 |
|  | 148683233 |
|  | 151555267 |
|  | 49726 |
|  | 52693 |
|  | 53549 |
|  | 74189907 |
|  | 149048033 |

---

|  |  |
| --- | --- |
| Swiss-Prot ID | IL6RA\_HUMAN |
|  | IL6RA\_MOUSE |
|  | Q3URV7\_MOUSE |
|  | A0N0L5\_HUMAN |
|  | A8KAE8\_HUMAN |

---

|  |  |
| --- | --- |
| Cell Localization | Basolateral cell membrane |
|  | Secreted |
|  | Membrane |
|  | Plasma membrane |

---

|  |  |
| --- | --- |
| Ensembl ID | ENSG00000160712 |
|  | ENSP00000357470.3 |
|  | ENST00000368485.8 |
|  | ENSP00000477739.1 |
|  | ENST00000622330.4 |
|  | ENSP00000340589.4 |
|  | ENST00000344086.8 |
|  | ENSMUSG00000027947 |
|  | ENSMUSP00000143541.1 |
|  | ENSMUST00000197679.4 |
|  | ENSMUSP00000029559.6 |
|  | ENSMUST00000029559.6 |
|  | ENSRNOG00000020811 |
|  | ENSRNOP00000028234.5 |
|  | ENSRNOT00000028234.7 |
|  | ENST00000368485.7 |
|  | ENSP00000357470 |
|  | ENST00000368485 |
|  | ENSP00000477739 |
|  | ENST00000622330 |
|  | ENSP00000340589 |
|  | ENST00000344086 |
|  | ENSMUSP00000029559 |
|  | ENSMUST00000029559 |
|  | ENSMUSP00000143541 |
|  | ENSMUST00000197679 |
|  | ENSRNOP00000028234 |
|  | ENSRNOT00000028234 |

---

|  |  |
| --- | --- |
| MGI ID | MGI:105304 |
|  | 105304 |

---

|  |  |
| --- | --- |
| RGD ID | 2902 |

---

|  |  |
| --- | --- |
| Unigene ID | Hs.135087 |
|  | Mm.2856 |
|  | Rn.1716 |
|  | Hs.709210 |
|  | Hs.591492 |
|  | Hs.695954 |

---

|  |  |
| --- | --- |
| Homologene ID | 474 |

---

|  |  |
| --- | --- |
| Shape | Stick-vertex |

---

|  |  |
| --- | --- |
| IPI ID | IPI00031171 |
|  | IPI00413309 |
|  | IPI00126188 |
|  | IPI00205182 |
|  | IPI00206288 |

---

|  |  |
| --- | --- |
| Homo sapiens Chromosome position | 1q21 |

---

|  |  |
| --- | --- |
| Ensembl Transcript ID | ENST00000368485 |
|  | ENST00000344086 |

---

|  |  |
| --- | --- |
| FunctionalClass | enzyme binding |
|  | interleukin-6 receptor |
|  | hematopoietin/interferon-class (D200-domain) cytokine receptor |
|  | receptor |
|  | interleukin receptor |

---

|  |  |
| --- | --- |
| Mus musculus Chromosome position | 3 42.1 cM |

---

|  |  |
| --- | --- |
| KEGG pathway | Cytokine-cytokine receptor interaction |
|  | Hematopoietic cell lineage |
|  | Jak-STAT signaling pathway |

---

|  |  |
| --- | --- |
| Rattus norvegicus Chromosome position | 2q34 |

---

|  |  |
| --- | --- |
| Ensembl Gene ID | ENSG00000160712 |

---
